# Pemafibrate decreases triglycerides and small, dense LDL, but increases LDL-C depending on baseline triglycerides and LDL-C in type 2 diabetes patients with hypertriglyceridemia: an observational study

**DOI:** 10.1186/s12944-021-01434-8

**Published:** 2021-02-20

**Authors:** Ichiro Komiya, Akira Yamamoto, Suguru Sunakawa, Tamio Wakugami

**Affiliations:** 1 Department of Internal Medicine, Okinawa Medical Hospital, 2310 Tsuhako-Nishihara, Sashiki, Nanjo, Okinawa 9011414 Japan; 2 Department of Diabetes and Endocrinology, Medical Plaza Daido Central, 123 Daido, Naha, Okinawa, 9020066 Japan; 3 Department of Cardiology, Medical Plaza Daido Central, 123 Daido, Naha, Okinawa 9020066 Japan

**Keywords:** Low density lipoprotein cholesterol, Pemafibrate, Small, Dense LDL, Triglycerides, Type 2 daibetes

## Abstract

**Background:**

Pemafibrate, a selective PPARα modulator, has the beneficial effects on serum triglycerides (TGs) and very low density lipoprotein (VLDL), especially in patients with diabetes mellitus or metabolic syndrome. However, its effect on the low density lipoprotein cholesterol (LDL-C) levels is still undefined. LDL-C increased in some cases together with a decrease in TGs, and the profile of lipids, especially LDL-C, during pemafibrate administration was evaluated.

**Methods:**

Pemafibrate was administered to type 2 diabetes patients with hypertriglyceridemia. Fifty-one type 2 diabetes patients (mean age 62 ± 13 years) with a high rate of hypertension and no renal insufficiency were analyzed. Pemafibrate 0.2 mg (0.1 mg twice daily) was administered, and serum lipids were monitored every 4–8 weeks from 8 weeks before administration to 24 weeks after administration. LDL-C was measured by the direct method. Lipoprotein fractions were measured by electrophoresis (polyacrylamide gel, PAG), and LDL-migration index (LDL-MI) was calculated to estimate small, dense LDL.

**Results:**

Pemafibrate reduced serum TGs, midband and VLDL fractions by PAG. Pemafibrate increased LDL-C levels from baseline by 5.3% (− 3.8–19.1, IQR). Patients were divided into 2 groups: LDL-C increase of > 5.3% (group I, *n* = 25) and < 5.3% (group NI, *n* = 26) after pemafibrate. Compared to group NI, group I had lower LDL-C (2.53 [1.96–3.26] vs. 3.36 [3.05–3.72] mmol/L, *P* = 0.0009), higher TGs (3.71 [2.62–6.69] vs. 3.25 [2.64–3.80] mmol/L), lower LDL by PAG (34.2 [14.5, SD] vs. 46.4% [6.5], *P* = 0.0011), higher VLDL by PAG (28.2 [10.8] vs. 22.0% [5.2], *P* = 0.0234), and higher LDL-MI (0.421 [0.391–0.450] vs. 0.354 [0.341–0.396], *P* < 0.0001) at baseline. Pemafibrate decreased LDL-MI in group I, and the differences between the groups disappeared. These results showed contradictory effects of pemafibrate on LDL-C levels, and these effects were dependent on the baseline levels of LDL-C and TGs.

**Conclusions:**

Pemafibrate significantly reduced TGs, VLDL, midband, and small, dense LDL, but increased LDL-C in diabetes patients with higher baseline TGs and lower baseline LDL-C. Even if pre-dose LDL-C remains in the normal range, pemafibrate improves LDL composition and may reduce cardiovascular disease risk.

## Background

Major efforts have been made to lower cholesterol (particularly LDL-C) in daily clinical practice [1]. In the case of increased triglycerides (TGs), however, there are insufficient active interventions for reducing TGs, as the TG-lowering effect of existing fibrates is insufficient [2] and the risk of adverse effects of drug combination is increased. In addition, consistent evidence that lowering TG levels reduces the risk of cardiovascular events is not currently available in randomized trials [2].

It has been reported that serum LDL-C levels and arteriosclerosis risk are generally positively correlated and that the decrease in risk due to LDL-C-lowering therapy is proportional to LDL-C reduction rate [1]. So-called statins (HMG-CoA reductase inhibitors) and proprotein convertase subtilisin/kexin type 9 (PCSK-9) inhibitors to reduce LDL-C by approximately half at maximum are used for the purpose of preventing arteriosclerotic diseases including cardiovascular disease (CVD) [3, 4]. At present, however, the residual risk of 50% or more has not been resolved, and indicators other than LDL-C level such as lower HDL-C and TG-rich lipoprotein (chylomicron remnants, VLDL remnants, or large VLDL etc.) have been regarded as therapeutic targets [5, 6]. A large-scale cohort study showed that the reduction of LDL-C was not so important for secondary prevention of CVD [7, 8]. Results from large-scale clinical trials have shown that hypertriglyceridemia carries a residual risk of cardiovascular events even with statin use [9, 10].

Pemafibrate (Palmodia®, a selective PPARα modulator), which was newly released in 2018, has a stronger TG-lowering effect than existing fibrate preparations and can be used in combination with statins [11–13]. PPARα, a nuclear receptor expressed mainly in the liver, is involved in regulating genes associated with lipid metabolism. Pemafibrate selectively regulates target genes involved in lipid metabolism among these PPARα-regulated genes [14]. The characteristic structure of pemafibrate enables alterations in gene transcription following recruitment of different cofactors [15].

There are many research reports that existing fibrates reduce LDL-C by about 10% [16, 17]. The present study clarified the relationship between lipoprotein fractions, LDL-C, and TGs before and after pemafibrate administration to Japanese type 2 diabetes patients with hypertriglyceridemia.

## Methods

### Patients and study procedures

The target subjects were outpatients with type 2 diabetes and lifestyle-related diseases visiting Medical Plaza Daido Central. Patients who were heavy drinkers were excluded from this retrospective study. Patients with eGFR less than 45 ml/min were also excluded because TG-rich lipoprotein and LDL-C accumulate in CKD, especially early stage CKD [18]. Seventy-two patients with type 2 diabetes were enrolled in this study, and 21 patients receiving statins, ezetimibe, or conventional fibrates were excluded, so 51 type 2 diabetes patients were recruited. All patients received pemafibrate 0.2 mg (0.1 mg twice daily) [11]. All patients did not change their exercise or dietary regimens including alcohol consumption, during the entire study period. Sodium-glucose cotransporter 2 inhibitors having lipid-improving activity were not administered during the study period.

Lipoprotein electrophoresis (polyacrylamide gel, PAG) was examined in 42 patients from − 8 weeks before administration to just before pemafibrate administration, and in 40 patients between 4 and 12 weeks after administration. PAG electrophoresis is covered by public health insurance and is often used as a routine diagnostic procedure for dyslipidemia in Japan. PAG electrophoresis revealed 4 lipoprotein fractions (HDL, VLDL, midband and VLDL). The LDL-migration index (LDL-MI) was calculated from the pattern of PAG electrophoresis according to a previous report [19]; that is, the PAG electrophoretic distance between the LDL and VLDL fractions was divided by that between the HDL and VLDL fractions. When this value was > 0.400, it was determined to be an increase in small, dense LDL (sd-LDL) [19, 20]. LDL-C was measured by the direct method using Metabolead® LDL-C (Hitachi Kasei Diagnostic Systems, Tokyo, Japan) [21].

Since TG levels are apt to fluctuate under the influence of diet [10], it was confirmed that fasting TG was > 1.69 mmol/L (150 mg/dL) by repeated measurements. Blood samples were collected after 9–12 h fasting. Serum lipids were monitored 4–8 weeks before administration. The average of the results at 8 weeks before (week − 8), at 4 weeks before (week − 4), and at start of pemafibrate (week 0) was used as the baseline value. Blood samples were analyzed every 4–8 weeks after pemafibrate, up to 24 weeks, and the average of the values was taken as the mean post-dose value, and the LDL-C increase rate was calculated using baseline and post-dose values. Assuming that the LDL-C increase rate varied among individual patients, 51 patients were divided into 2 groups, the increased LDL-C group (group I) and the no LDL-C increase group (NI group), with the median LDL-C increase rate as the boundary. Results of basal clinical parameters were shown as the mean (SD). Results of lipid and liver function tests were shown as the median (25–75% quartile, IQR) due to non-parametric distribution.

### Statistical analysis

Statistical analysis was performed by t-test, paired t-test, Wilcoxon signed-rank test, Mann-Whitney U test, Kruskal-Wallis test, χ^2^ test. Linear regression analysis was applied using least-square method. Multivariate regression analysis was performed using 9 baseline variables (LDL-C, TGs, HDL-C, Non − HDL-C, HDL, LDL, midband, VLDL and LDL-MI) to examine determinants for the LDL-C increase rate. JMP for Windows version 12 software (SAS Institute Japan; Tokyo, Japan) was used for statistical analyses. *P* values of < 0.05 were considered statistically significant.

## Results

### Changes in lipid profile after pemafibrate administration

The clinical background of 51 target patients was shown in Table 1. The mean age was 62.2 (SD 12.9) years, the BMI was 26.6 (3.7) kg/m^2^, and the proportion of males was 55%. The average HbA1c was 7.4% (1.4), and the average eGFR was 71.9 (22.3) ml/min/1.73 m^2^. Hypertension and CVD/stroke complications were 59 and 18%, respectively.

**Table 1:** Baseline characteristics of all patients and patients with/ without an increase in LDL-C after pemafibrate

Characteristic	Total	Increased LDL-C group (group I)^a^	No LDL-C increase group (group NI)^b^	P*
Cases no. (%)	51(100)	25 (49)	26 (51)	
Cases of LDL-C increase, no. (%)	35 (69)	25 (100)	10 (38)	
% increase in LDL-C, median (IQR)	5.3 (−3.8–19.1)	19.1 (13.3–56.3)	− 3.7 (−13.0–1.2)	
Males, no. (%)	28 (55)	17 (68)	11 (42)	0.0653
Age, mean (SD), years	62.2 (12.9)	60.7 (13.2)	63.7 (12.7)	0.4168
BMI, mean (SD), kg/m^2^	26.6 (3.7)	27.1 (4.0)	26.1 (3.4)	0.3496
HbA1c, mean (SD), %	7.4 (1.4)	7.2 (1.3)	7.5 (1.5)	0.5568
eGFR, mean (SD), mL /min/1.73 m^2^	71.9 (22.3)	74.8 (23.4)	69.2 (21.3)	0.3732
Complications				
Hypertension, no. (%)	30 (59)	14 (56)	16 (62)	0.6879
CVD/stroke, no. (%)	9 (18)	6 (24)	3 (12)	0.2432
Treatment for diabetes				
OHA, no. (%)	36 (71)	16 (64)	20 (77)	0.4685
Insulin + OHA, no. (%)	4 (8)	3 (12)	1 (4)	

^a^% increase in LDL-C > 5.3%; ^b^% increase in LDL-C < 5.3%. *P* < 0.05 was considered statistically significant (χ^2^ test or t-test). *Statistical analysis between increased LDL-C and no LDL-C increase groups using the χ^2^ test or the t-test. *BMI* Body mass index, *HbA1c* Glycated hemoglobin, *eGFR* Estimated glomerular filtration rate, *LDL-C* Low density lipoprotein cholesterol, *IQR* Inter-quartile range, *OHA* ORAL hypoglycemic agents.

The LDL-C increase rate after pemafibrate administration varied from patient to patient. The LDL-C increase rate fluctuated significantly from − 27.8 to 125.6% depending on the case before and after administration with pemafibrate. Thirty-five of 51 patients (69%) showed LDL-C increase. Since the median of LDL-C increase rate in 51 target patients was 5.3% (IQR − 3.8-19.1), patients with an LDL-C increase rate of > 5.3% were defined as the increased LDL-C group (group I; 25 cases) and the median LDL-C increase rate in group I after administration was 19.1% (IQR 13.3–56.3). Those with an LDL-C increase rate < 5.3% were defined as the no LDL-C increase group (NI group; 26 cases) and the median LDL-C increase rate in group NI after administration was − 3.7% (− 13.0–1.2) (Table 1). As shown in Table 1, there were no significant difference in basal clinical background between the 2 groups.

### Comparison of lipid profiles between 2 groups after pemafibrate administration

Table 2 showed the changes in TG levels before and after administration (week − 8 to week 24). Pemafibrate significantly reduced TGs during the administration period of 24 weeks (*P* < 0.0001, Kruskal-Wallis test). Comparing TG between baseline and post-dose, the median TG decreased from 3.30 (IQR 2.63–4.76) to 2.15 (1.72–2.44) mmol/L after administration (*P* < 0.0001, Wilcoxon signed-rank test). The median LDL-C increased slightly from 3.10 (IQR 2.40–3.59) to 3.19 (2.74–3.70) mmol/L after administration (*P* = 0.0170). HDL-C increased by approximately 0.10 mmol/L during 24-week administration, and the difference was significant (*P* < 0.0001). Moreover, non − HDL-C was reduced after pemafibrate administration (4.84 [4.32–5.28] vs. 4.14 [3.52–4.58]) mmol/L, *P* < 0.0001).

**Table 2:** Changes in lipid profile after pemafibrate treatment

Characteristic	Weeks after pemafibrate treatment										Pemafibrate treatment		
	Week − 8	Week − 4	Week 0	Week 4	Week 8	Week 12	Week 16	Week 20	Week 24	P*	Baseline^a^	Post-dose^b^	P**
**TG**, median (IQR),	mmol/L												
Total	3.01 (2.50–4.08)	3.49 (2.60–5.43)	3.60 (2.69–5.44)	2.19 (1.55–2.47)	2.11 (1.47–2.42)	1.99 (1.42–2.87)	2.27 (1.72–2.81)	2.70 (2.01–3.59)	2.08 (1.75–2.69)	< 0.0001	3.30 (2.63–4.76)	2.15 (1.72–2.44)	< 0.0001
Increased LDL-C group (group I)	3.25 (2.47–6.27)	4.52 (2.63–6.11)	4.03 (2.60–7.24)	2.22 (1.56–2.47)	2.07 (1.40–2.45)	2.00 (1.85–3.07)	2.27 (1.73–3.10)	2.74 (2.04–4.21)	1.90 (1.59–2.83)	< 0.0001	3.71 (2.62–6.69)	2.11 (1.61–2.57)	< 0.0001
No LDL-C increase group (group NI)	2.90 (2.51–3.77)	3.01 (2.31–4.03)	3.26 (2.77–4.08)	2.10 (1.50–2.54)	2.17 (1.68–2.42)	1.70 (1.26–2.50)	2.22 (1.64–2.69)	2.57 (1.82–3.10)	2.24 (1.82–2.69)	< 0.0001	3.25 (2.64–3.80)	2.20 (1.92–2.42)	< 0.0001
P***	0.3946	0.0683	0.1178	0.5890	0.6870	0.3952	0.7561	0.5934	0.7648		0.1495	0.6241	
**LDL-C**, median (IQR),	mmol/L												
Total	3.10 (2.61–3.59)	3.00 (2.23–3.47)	3.08 (2.30–3.59)	2.84 (2.33–3.41)	3.15 (2.64–3.72)	2.72 (2.15–3.36)	3.13 (2.59–3.52)	2.72 (1.97–3.65)	2.92 (2.59–3.57)	0.8679	3.10 (2.40–3.59)	3.19 (2.74–3.70)	0.0170
Increased LDL-C group (group I)	2.66 (2.02–3.21)	2.66 (1.91–3.52)	2.51 (1.71–3.13)	3.21 (2.25–3.49)	3.28 (2.72–4.19)	3.10 (1.91–3.67)	3.00 (2.46–3.75)	3.31 (1.86–3.96)	3.39 (2.87–4.09)	0.0483	2.53 (1.96–3.26)	3.31 (2.77–4.11)	< 0.0001
No LDL-C increase group (group NI)	3.26 (2.92–3.78)	3.26 (2.82–3.47)	3.52 (2.87–3.75)	2.69 (2.33–2.84)	2.92 (2.64–3.41)	2.59 (2.22–3.05)	3.15 (2.72–3.44)	2.64 (2.28–3.21)	2.74 (2.56–3.10)	0.0157	3.36 (3.05–3.72)	3.03 (2.66–3.44)	0.0022
P***	0.0024	0.0759	0.0008	0.4414	0.1583	0.4616	0.7042	0.8415	0.0603		0.0009	0.2418	
**HDL-C**, median (IQR),	mmol/L												
Total	1.27 (1.01–1.45)	1.22 (0.98–1.42)	1.22 (1.01–1.42)	1.22 (1.09–1.45)	1.37 (1.16–1.53)	1.42 (1.14–1.55)	1.29 (1.06–1.47)	1.22 (0.98–1.55)	1.27 (1.01–1.55)	0.5147	1.22 (1.03–1.40)	1.32 (1.14–1.53)	< 0.0001
Increased LDL-C group (group I)	1.16 (0.93–1.37)	1.22 (0.96–1.32)	1.14 (0.85–1.32)	1.14 (1.06–1.27)	1.37 (1.06–1.50)	1.37 (1.22–1.45)	1.22 (0.91–1.50)	1.19 (0.96–1.55)	1.27 (1.03–1.66)	0.2396	1.16 (0.93–1.29)	1.29 (1.09–1.53)	< 0.0001
No LDL-C increase group (group NI)	1.29 (1.14–1.55)	1.29 (1.03–1.58)	1.32 (1.06–1.50)	1.42 (1.11–1.47)	1.37 (1.16–1.60)	1.45 (0.98–1.71)	1.34 (1.11–1.47)	1.24 (1.09–1.55)	1.29 (1.09–1.53)	0.9936	1.32 (1.06–1.50)	1.34 (1.14–1.55)	0.0258
P***	0.0465	0.1829	0.0194	0.1589	0.5368	0.9096	0.6165	0.8413	1.0000		0.0267	0.5090	
**Non − HDL-C**, median	(IQR),	mmol/L											
Total	4.71 (4.37–5.15)	4.55 (4.34–5.72)	4.71 (4.27–5.43)	3.52 (3.10–4.37)	4.27 (3.52–4.78)	3.80 (3.10–4.34)	4.14 (3.72–4.45)	4.03 (3.59–4.47)	3.96 (3.62–4.31)	< 0.0001	4.84 (4.32–5.28)	4.14 (3.52–4.58)	< 0.0001
Increased LDL-C group (group I)	4.73 (4.09–5.46)	4.65 (3.98–6.52)	4.55 (4.01–5.33)	3.85 (3.26–4.76)	4.45 (3.52–4.99)	4.16 (3.28–4.53)	3.98 (3.67–4.55)	3.98 (3.57–4.78)	4.06 (3.72–4.99)	0.1581	4.86 (4.03–5.35)	4.24 (3.41–4.84)	0.0063
No LDL-C increase group (group NI)	4.71 (4.40–5.04)	4.55 (4.34–5.59)	4.84 (4.40–5.66)	3.34 (2.82–3.70)	4.19 (3.39–4.63)	3.62 (2.84–3.98)	4.22 (3.75–4.42)	4.09 (3.08–4.34)	3.75 (3.52–4.11)	< 0.0001	4.76 (4.45–5.20)	4.06 (3.57–4.50)	< 0.0001
P***	0.7853	0.9613	0.2362	0.2466	0.2472	0.3356	0.6662	0.8415	0.1653		0.6834	0.5220	

*P* < 0.05 was considered statistically significant. ^a^Mean value of 3 measurements before pemafibrate; ^b^Mean value of 6 measurements after pemafibrate. *, Statistical analysis was done using the Kruskal-Wallis test. **, Statistical analysis was done using Wilcoxon signed-rank test. ***, Statistical analysis between groups I and NI was done using the Mann-Whitney U test. *IQR* Inter-quartile range, *TG* Triglycerides, *LDL-C* Low density lipoprotein cholesterol, *HDL-C* High density lipoprotein cholesterol

**Fig. 1:**
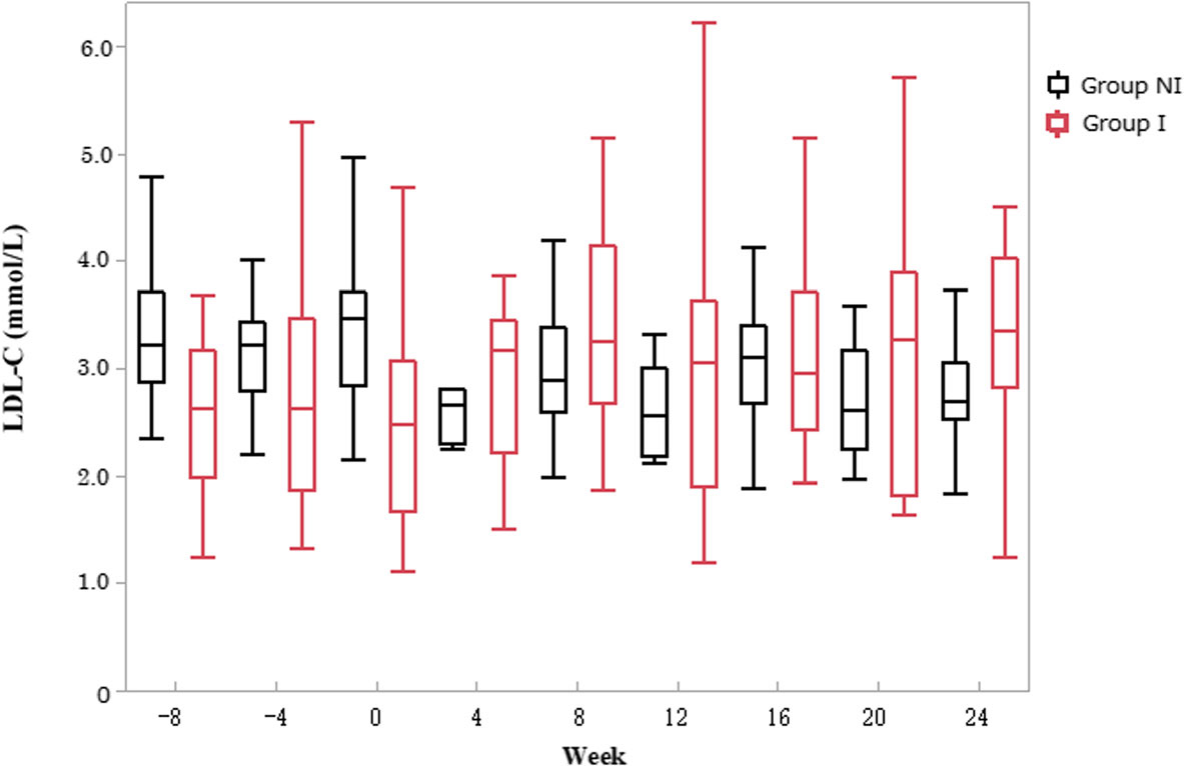
Profile of LDL-C between groups before and after pemafibrate administration. Median and IQR of LDL-C in group I are shown in red boxplots and those in group NI are shown in black boxplots

As mentioned above, LDL-C increased notably after administration in group I; therefore, lipid profiles were compared between groups I and NI in next step. As shown in Table 2 and Fig. 1, the increasing tendency in LDL-C continued throughout the administration period (*P* = 0.0483, Kruskal-Wallis test). Comparing LDL-C levels in group I between baseline and post-dose, the median LDL-C increased from 2.53 (IQR 1.96–3.26) to 3.31 (2.77–4.11) mmol/L after administration (*P* < 0.0001, Wilcoxon signed-rank test). Group I was also characterized by high TGs before administration compared with group NI (3.71 [2.62–6.69] vs. 3.25 [2.64–3.80] mmol/L), but the difference in TG levels between the 2 groups disappeared after pemafibrate. Non − HDL-C was slightly higher in group I than in group NI (4.86 [4.03–5.35] vs. 4.76 [4.45–5.20] mmol/L) and decreased in both groups after pemafibrate administration.

Table 3 showed the results of lipoprotein fraction analyses and LDL-MI in PAG electrophoresis. PAG electrophoresis revealed 4 lipoprotein fractions (HDL, VLDL, midband and VLDL) but there were cases where the midband fraction does not exist. Before pemafibrate administration, the midband fraction was high in both groups. The increase of midband suggests the increases of intermediate-density lipoprotein, VLDL remnant or remnant-like particle cholesterol, and relates with CVD [22]. The VLDL fraction was higher in group I than in group NI (28.2 [SD 10.8] vs. 22.0 [5.2], *P* = 0.0234, t-test). Furthermore, the LDL fraction was lower in group I than group NI (34.2 [14.5] vs. 46.4% [6.5], *P* = 0.0011). Lipoprotein PAG electrophoresis revealed a decrease in the VLDL fraction after pemafibrate administration but did not indicate the complete disappearance of the midband fraction in either group. The LDL fraction in group I increased from 34.2 [14.5] to 47.8% [10.9] after pemafibrate (*P* < 0.002). However, there was no difference in lipoprotein fractions between the groups after pemafibrate administration (Table 3).

**Table 3:** Results of analyses of lipoprotein fraction and LDL-MI after pemafibrate treatment

Characteristic	Baseline			Post-dose		
	Increased LDL-C group (group I) (*n* = 21)	No LDL-C increase group (group NI) (*n* = 21)	P*	Increased LDL-C group (group I) (*n* = 20)	No LDL-C increase group (group NI) (*n* = 20)	P*
Lipoprotein fraction (PAG electrophoresis)						
**HDL**, mean (SD), %	21.0 (7.8)	19.0 (5.2) **	0.3358	20.7 (5.6)	23.6 (5.2)	0.0907
**LDL**, mean (SD), %	34.2 (14.5)***	46.4 (6.5)	0.0011	47.8 (10.9)	44.9 (5.5)	0.2867
**Midband**, mean (SD), %	18.5 (8.5)	12.5 (6.4)	0.0141	14.7 (6.6)	14.4 (8.8)	0.9034
**VLDL**, mean (SD), %	28.2 (10.8) ***	22.0 (5.2) **	0.0234	17.1 (6.3)	17.3 (6.2)	0.9399
**LDL-MI**, median (IQR)	0.421 (0.391–0.450) ****	0.354 (0.341–0.396)	< 0.0001	0.367 (0.344–0.389)	0.348 (0.331–0.380)	0.2339

*P*<0.05 was considered statistically significant. *, Statistical analysis between 2 groups was done using the t-test or Mann-Whitney U test. **, *P*<0.02 *vs*. group NI at post-dose (t-test). ***, *P*<0.002 *vs*. group I at post-dose (t-test). ****, *P*=0.0002 *vs*. group I at post-dose (Mann-Whitney U test). *PAG* Polyacrylamide gel, *LDL-MI* LDL migration index, *LDL-C* Low density lipoprotein cholesterol, *IQR* Inter-quartile range, *HDL* High density lipoprotein, *LDL* Low density lipoprotein, *VLDL* Very-low density lipoprotein

**Fig. 2:**
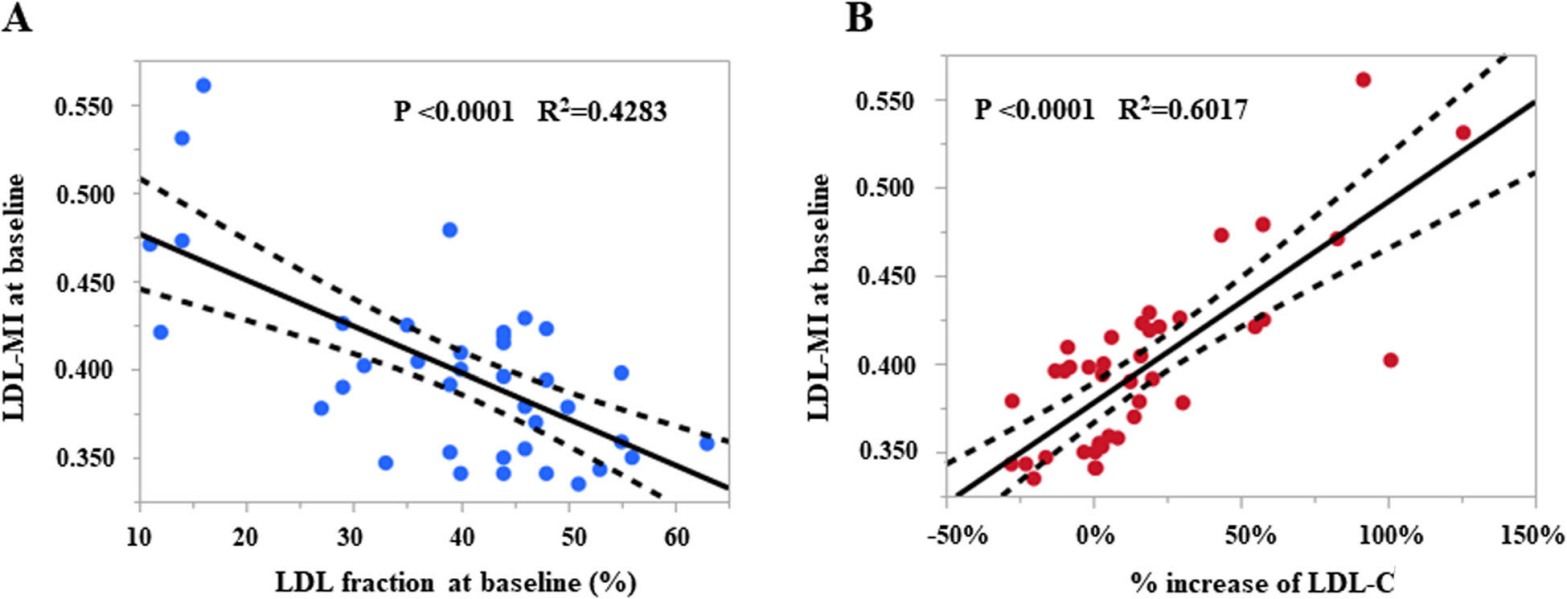
A Relationship between LDL-C and LDL-MI before pemafibrate. The regression line and its 95% confidence interval are shown (R^2^ = 0.4283, *P* < 0.0001, least squares method). B Relationship between percent increase in LDL-C and LDL-MI before pemafibrate. The regression line and its 95% confidence interval are shown (R^2^ = 0.6017, *P* < 0.0001)

The LDL-MI before pemafibrate in group I was 0.421 (0.391–0.450), exceeding 0.400, which was significantly different from the NI group (0.354 [0.341–0.396], *P* < 0.0001, Mann-Whitney U test). These results suggest that sd-LDL increased in group I. The LDL-MI in group I dropped to 0.367 (0.344–0.389) after pemafibrate (*P* = 0.0002), but it was > 0.400 in some cases. However, the LDL-MI was almost the same in both groups after pemafibrate. Figure 2a showed the correlation between LDL-MI and LDL fraction before pemafibrate in all measured cases. A significant inverse correlation was observed (*R*^2^ = 0.4283, *P* < 0.0001, least squares). Figure 2b showed the correlation between LDL-MI before pemafibrate and LDL-C increase rate before and after pemafibrate. A significant positive correlation was observed (*R*^2^ = 0.6017, *P* < 0.0001).

As shown in Table 4, a stepwise multivariate regression analysis was performed using 9 baseline variables, and 5 baseline variables (LDL-MI, TGs, LDL-C, HDL-C and Non − HDL-C) were finally included in the multivariate regression model as independent variables that interacted with LDL-C increase rate. Baseline LDL-C levels significantly correlated with LDL-C increase rate (β = − 0.8911, *P* < 0.0001). Baseline LDL-MI (β = 0.5176, *P* < 0.0001), Non − HDL-C (β = 0.7649, *P* = 0.0001) and TGs (β = 0.5002, *P* = 0.0057) positively correlated with LDL-C increase rate.

**Table 4:** Multivariate regression analysis between LDL-C increase rate and baseline lipid parameters

Variables	β (standardized coefficient)	*P*
**Baseline LDL-C**	**−**0.8911	<0.0001
**Baseline TGs**	0.5002	0.0057
**Baseline Non − HDL-C**	0.7649	0.0001
**Baseline LDL-MI (PAG)**	0.5176	<0.0001
**Baseline HDL-C**	0.1413	0.1341
**Baseline HDL (PAG)**	NI	–
**Baseline LDL (PAG)**	NI	–
**Baseline Midband (PAG)**	NI	–
**Baseline VLDL (PAG)**	NI	–

Statistical analysis by stepwise multiple regression was performed to examine determinant factors for LDL-C increase rate. *P* < 0.05 was considered statistically significant. *NI* Not included in the model, *PAG* Polyacrylamide gel

### Changes in liver function tests

No patients exhibited worsened liver function tests during pemafibrate administration. Liver function tests showed 21% decrease in ALT (24 [17–37] vs. 19 [16–30] IU/L, *P* = 0.0010, Wilcoxon signed-rank test). and 16% decrease in γGTP (37 [26–86] vs. 31 [19–54] IU/L, *P* < 0.0001). Baseline levels of ALT and γGTP were slightly higher in group I than in group NI. The improvement rates of ALT and γGTP in group I were higher than those in group NI, but no statistical difference between 2 groups was shown. HbA1c did not change at all throughout the 24 weeks of pemafibrate administration (Table 5).

**Table 5:** Changes in liver function and HbA1c after pemafibrate treatment

Characteristic	Weeks after pemafibrate treatment										Pemafibrate treatment		
	Week −8	Week −4	Week 0	Week 4	Week 8	Week 12	Week 16	Week 20	Week 24	P*	Baseline^a^	Post-dose^b^	P**
**AST,** median (IQR),	IU/L												
Total	21 (18–29)	22 (18–30)	23 (19–33)	21 (18–34)	23 (18–31)	24 (21–44)	23 (19–29)	22 (19–33)	21 (19–29)	0.9212	23 (18–31)	23 (19–31)	0.9139
Increased LDL-C group (group I)	23 (19–34)	26 (21–33)	23 (20–36)	19 (18–32)	20 (17–33)	26 (21–48)	23 (18–40)	23 (20–43)	20 (19–22)	0.6619	23 (19–34)	21 (18–34)	0.3833
No LDL-C increase group (group NI)	20 (17–27)	20 (17–29)	20 (18–29)	21 (19–35)	24 (19–28)	23 (21–40)	24 (20–28)	21 (17–49)	23 (16–34)	0.9440	20 (18–28)	25 (20–29)	0.6184
P***	0.2611	0.1970	0.1894	0.4655	0.3690	0.6480	0.9545	0.5388	0.8421		0.2273	0.4646	
**ALT**, median (IQR),	IU/L												
Total	22 (18–36)	26 (18–37)	23 (18–40)	19 (16–29)	20 (17–27)	19 (17–58)	21 (16–31)	18 (15–23)	17 (15–24)	0.1297	24 (17–37)	19 (16–30)	0.0010
Increased LDL-C group (group I)	28 (19–40)	30 (19–39)	31 (19–41)	19 (17–28)	20 (15–38)	19 (16–62)	23 (17–38)	19 (15–33)	18 (14–25)	0.1802	31 (21–40)	19 (16–34)	0.0040
No LDL-C increase group (group NI)	22 (17–29)	23 (16–36)	20 (17–29)	19 (15–38)	20 (17–25)	17 (17–53)	21 (16–30)	18 (14–53)	17 (15–24)	0.9352	22 (17–29)	21 (17–26)	0.0909
P***	0.1606	0.1605	0.0582	1.0000	0.9269	0.9091	0.5141	1.0000	0.8427		0.0520	0.9918	
**γGTP**, median (IQR),	IU/L												
Total	34 (26–94)	46 (28–83)	39 (24–92)	34 (21–48)	30 (18–47)	43 (31–74)	28 (15–57)	34 (20–57)	33 (20–65)	0.0656	37 (26–86)	31 (19–54)	<0.0001
Increased LDL-C group (group I)	43 (31–98)	67 (31–110)	53 (28–102)	39 (29–60)	32 (22–53)	53 (32–80)	37 (14–71)	40 (29–67)	38 (25–71)	0.2944	48 (28–101)	34 (26–75)	< 0.0001
No LDL-C increase group (group NI)	31 (20–77)	36 (24–63)	32 (18–54)	20 (12–49)	23 (17–34)	31 (14–44)	25 (16–47)	18 (13–46)	31 (15–49)	0.4730	30 (19–62)	26 (17–43)	<0.0001
P***	0.0975	0.0643	0.0382	0.1118	0.1074	0.0471	0.2486	0.0570	0.1392		0.0600	0.0992	
**HbA1c**, mean (SD),	%												
Total	7.5 (1.5)	7.7 (1.5)	7.4 (1.6)	7.6 (1.2)	7.4 (1.6)	7.2 (1.4)	7.2 (1.3)	7.9 (1.4)	7.1 (1.9)	0.7897	7.4 (1.4)	7.3 (1.5)	0.0502
Increased LDL-C group (group I)	7.6 (1.7)	7.5 (1.3)	7.2 (1.1)	7.4 (1.3)	7.3 (1.3)	7.0 (1.6)	7.3 (1.4)	7.7 (1.8)	6.7 (1.4)	0.8251	7.2 (1.3)	7.1 (1.3)	0.1510
No LDL-C increase group (group NI)	7.5 (2.0)	8.0 (1.6)	7.5 (2.0)	7.8 (1.1)	7.5 (1.9)	7.4 (1.1)	7.2 (1.3)	8.1 (0.8)	7.4 (2.3)	0.9534	7.5 (1.5)	7.4 (1.6)	0.1561
P***	0.7866	0.3344	0.5476	0.5551	0.6126	0.6060	0.7508	0.6851	0.3621		0.5568	0.4819	

*P*<0.05 was considered statistically significant. *, Statistical analysis was done using the Kruskal-Wallis test or ANOVA. ^a^Mean value of 3 measurements before pemafibrate; ^b^Mean value of 6 measurements after pemafibrate. **, Statistical analysis was done using Wilcoxon signed-rank test or paired t-test. ***, Statistical analysis between 2 groups I and NI was done using the Mann-Whitney U test or t-test. *LDL-C* Low density lipoprotein cholesterol, *HbA1c* Glycated hemoglobin

### Adverse effects

Of the 51 cases, one patient exhibited muscle pain symptoms, but no significant increase in CPK was observed.

## Discussion

This study demonstrated the long-term effect of pemafibrate on LDL-C levels for 24 weeks in patients with type 2 diabetes with hypertriglyceridemia. Pemafibrate 0.2 mg (0.1 mg twice daily) significantly reduced serum TGs and VLDL by PAG electrophoresis and slightly increased HDL-C following administration to type 2 diabetes patients with hypertriglyceridemia. Although LDL-C levels were not considerably altered, pemafibrate for 24 weeks increased LDL-C levels by 5.9% as median. The increase in LDL-C was stably maintained over 24 weeks. LDL-C levels decreased in only 31% (16 of 51) patients after pemafibrate. Furthermore, It was demonstrated for the first time that the increase in LDL-C after pemafibrate had lower LDL-C, higher TGs, higher Non − HDL-C, higher sd-LDL and higher midband at baseline.

It should be noted that there was a considerable number of cases of increased LDL-C in this study during treatment with pemafibrate. Previous study showed that the baseline TGs and LDL-C were key determinants of the changes in LDL-C [12]. However, the existence of an increased LDL-C was briefly mentioned but not discussed as the main issue in previous studies, including patients with dyslipidemia other than diabetes [11–13, 23–25]. Since present study only targets diabetes patients with hypertriglyceridemia, it is conceivable that the rate of increase in LDL-C was higher than in previous reports. Compared to group NI, group I had lower LDL-C (2.53 vs. 3.36 mmol/L), higher TGs (3.71 vs. 3.25 mmol/L), lower LDL fraction by PAG electrophoresis (34.2 vs. 46.4%), higher midband fraction (18.5 vs. 12.5%), and higher LDL-MI (0.421 vs. 0.354) at baseline. It was reported previously that bezafibrate slightly increased the LDL-C levels from 124 ± 37 mg/dL (3.21 ± 0.96 mmol/L) to 126 ± 31 mg/dl (3.26 ± 0.80 mmol/L) in diabetes patients, and LDL-C increase rate varied according to the baseline LDL-C level, with a significant increase in < 120 mg/dL (3.10 mmol/L) of LDL-C [26]. It was reported that a similar increase effect of LDL-C was brought by weight loss in hypertriglyceridemic patients [27].

This relationship between baseline TGs and LDL-C can be explained by the precursor-product relationship between VLDL and LDL, LDL is produced as an ultimate product of the lipolytic conversion of VLDL [28]. Fibrates, including pemafibrate, activate lipoprotein lipase (LPL) and hepatic triglyceride lipase to promote the catabolism TG-rich lipoprotein and also attenuate VLDL synthesis to reduce serum TGs [29]. It is assumed that LDL and HDL are produced during the catabolism of the TG-rich lipoprotein [27, 28]. Since pemafibrate has a stronger TG-rich lipoprotein catabolism than existing fibrates, it is presumed that pemafibrate increases LDL-C and changes LDL-C composition [11–13, 30]. Pemafibrate has been reported to increase the size and decrease the number of LDL particles [24]. The increase in LDL-C after pemafibrate treatment suggests the actual improvement in lipoprotein metabolism. On the other hand, the increase in LDL-C levels with TG reduction by pemafibrate might lead clinicians to question the clinical efficacy of the treatment. Further studies are needed to define the mechanisms underlying the variability of the effects of pemafibrate on LDL-C.

The baseline LDL-MI in group I exceeded 0.400, suggesting that sd-LDL increased [30]. This study thus revealed that baseline sd-LDL increases and significantly improves after pemafibrate administration. Higher baseline TGs and lower baseline LDL-C appear to be involved in the baseline sd-LDL increase and post-dose LDL-C increase. The ARIC study reported that the risk of CVD was associated with an increase in sd-LDL-C rather than large buoyant LDL-C [31]. In a study of ischemic heart disease in elderly Japanese men, patients with high levels of sd-LDL had increased risk of CVD events over the next 5 years [32]. In the present study, CVD/stroke complications were not higher in group I, but the number of cases was too small to draw conclusions.

Higher values of sd-LDL occur when both non − HDL-C and TGs are high [33]. As the present study showed an inverse correlation between baseline LDL-MI and baseline LDL fraction by PAG electrophoresis, it was suggested that an increase in sd-LDL and a decrease in the LDL fraction are synchronized phenomenon associated with lipid metabolism in patients with type 2 diabetes. There was a positive correlation between baseline LDL-MI and the LDL-C increase rate. This strongly suggests that pemafibrate decreases sd-LDL and increases large buoyant LDL in type 2 diabetes patients.

PCSK9 inhibitors, monoclonal antibodies that bind to free PCSK9, have potent effect on LDL-C reduction, and are recommended to use in patients with high CVD risk or familial hypercholesterolemia [34]. Whether or not PCSK9 inhibitors reduce CVD events is not yet to be clarified [35]. Moreover, PCSK9 inhibitors seem to be less efficient lowering sd-LDL [36], different from the pemafibrate effect.

Hypertriglyceridemia is defined as a fasting TG value of ≥1.69 mmol/L (150 mg/dL). As in hypercholesterolemia (mainly increased LDL-C), hypertriglyceridemia has been epidemiologically associated with many atherosclerotic diseases including CVD [37]. However, unlike cholesterol, TGs do not accumulate in atherosclerotic plaques on the walls of blood vessels, and TGs per se do not promote atherosclerosis [38]. In general, when serum TG concentrations rise, the cholesterol contained in TG-rich lipoprotein increases and total cholesterol concentrations also rise [39]. In patients with hypertension and/or insulin resistance, the metabolism of lipoproteins is delayed and they remain in the blood circulation for a variety of reasons [40]. The retained remnants are taken up by macrophages, and cholesterol accumulates in atherosclerotic lesions [41].

It is well known that VLDL and LDL are apo B-containing lipoproteins associated with arteriosclerosis. Lowering LDL-C by 40 mg/dL (1.03 mmol/L) has been reported to reduce the risk of cardiovascular events by 20% [6]. Assuming that all apo B-containing particles have almost the same atherogenic effect [6], the TG value needs to be reduced by 5 times that of LDL-C, approximately 200 mg/dL (2.26 mmol/L), from a simple interpretation of Friedewald’s formula. However, the TG-lowering effect of existing fibrates is not significant, making it impossible to significantly reduce the risk of major cardiovascular events [23]. On the other hand, in present study, pemafibrate significantly decreased TGs from 3.30 to 2.15 mmol/L in all cases and from 3.71 to 2.11 mmol/L in patients with higher LDL-C increase rate. It was concluded that this significant reduction in TGs caused a decrease in large VLDL and a change in LDL composition [42]. This study suggested that pemafibrate increased LDL-C not by increasing the particle number of LDL, but rather by increasing the cholesterol content of LDL.

In hypertriglyceridemia, VLDL cholesterol and/or non − HDL-C are increased, which has atherosclerosis-inducing properties similar to or higher than LDL cholesterolemia [43]. According to current guidelines for arteriosclerosis, non − HDL-C should be evaluated instead of LDL-C in cases such as severe hypertriglyceridemia [5]. The LDL-C value is said to underestimate the cardiovascular risk. When the non − HDL-C level is high, cholesterol lowering therapy is prioritized, as in LDL cholestrolemia [5, 44]. Non − HDL-C was certainly high before pemafibrate and decreased after administration in this study.

### Study strength and limitations

There are several study strengths in this study. First, the reliable LDL-C direct assay, a Metabolead LDL-C® (Hitachi Kasei Diagnostic Systems), could be used for LDL-C estimation, and the results were consistent with the lipoprotein PAG electrophoresis results. Moreover, this direct method has already been shown to be consistent with ultracentrifugation, unless the TGs exceed 11.29 mmol/L (1,000 mg/dL) [21]. There were no patients with a fasting TG level of 11.29 mmol/L or higher in this study, therefore, any effect of hypertriglyceridemia on the LDL-C assay (false low value) could be ruled out. Second, serum lipids were followed every 4–8 weeks for 24 weeks to evaluate lipids with variable values. Measuring the lipid profile several times is more accurate than simply assessing it before and after pemafibrate administration. Finally, PAG electrophoresis, which is a simple and inexpensive method, was used for the estimation of sd-LDL and lipoprotein fractions. Thus, in some diabetes cases, LDL-C was relatively low before pemafibrate and LDL-C increased after pemafibrate, demonstrating that the composition of LDL was significantly changed due to the TG-rich lipoprotein lowering effect of pemafibrate.

This study has several limitations. First, instead of directly measuring sd-LDL, determination of LDL-MI by PAG electrophoresis was used as a substitute. However, many reports indicated that the results of both are strongly correlated [19, 20]. Second, there were not many target patients in this study, and because of the retrospective nature, results of lipoprotein PAG electrophoresis were not obtained in several patients. However, statistically significant results were obtained despite the small number of patients. It would be needed to increase the number of target patients in the future. Finally, this study did not show results for serum apolipoproteins such as apo B and apo E. It cannot be ruled out that present study may include patients with type III hyperlipidemia [45]. As the patients had a high proportion of combined hyperlipidemia, it might be close to the “lipoprotein abnormality similar to type III hyperlipidemia” reported by Matsuzawa et al. [46]. Cardiovascular events are frequently observed in “lipoprotein abnormalities similar to type III hyperlipidemia”, so the future risk of CVD/stroke complications in the examined cases should be followed closely [13].

## Conclusion

The superiority of pemafibrate allows control of serum TG levels and sd-LDL, which were previously inadequate with conventional treatment [2, 11, 22]. At the same time, there were cases in which LDL-C levels fluctuate markedly before and after pemafibrate administration. It was also shown that measurement of LDL-C by the direct assay instead of the recommended measurement of non − HDL-C is useful in the pathologic evaluation of type 2 diabetes patients with high TGs. It is necessary to review recommendations in the arteriosclerosis guideline [5]. Moreover, the direct LDL-C assay combined with lipoprotein PAG electrophoresis enables easy evaluation of TG-rich lipoprotein. Even in statin-treated type 2 diabetes patients whose LDL-C remains within the therapeutic range, clear increases in sd-LDL with hypertriglyceridemia are inherent [31], pemafibrate or ezetimibe, which have a TG-rich lipoprotein-lowering effect, might be considered as drugs for add-on therapy [47]. The above data suggest a need to review the diagnostic indices and control standards for lipids in patients with type 2 diabetes.

## Data Availability

The collection of data that supports the findings in this study is available from Medical Plaza Daido Central. Data are available from the authors upon reasonable request and with permission of Medical Plaza Daido Central.

## References

[1] Mihaylova B, Emberson J, Blackwell L, Keech A, Simes J, Cholesterol Treatment Trialistsʼ (CTT) Collaborators (2012). The effects of lowering LDL cholesterol with statin therapy in people at low risk of vascular disease: meta-analysis of individual data from 27 randomised trials. Lancet.

[2] Keech A, Simes RJ, Barter P, Best J, Scott R, Taskinen MR (2005). FIELD study investigators. Effects of long-term fenofibrate therapy on cardiovascular events in 9795 people with type 2 diabetes mellitus (the FIELD study): randomised controlled trial. Lancet..

[3] Baigent C, Blackwell L, Emberson J, Holland LE, Reith C, Cholesterol Treatment Trialists’ (CTT) Collaboration (2010). Efficacy and safety of more intensive lowering of LDL cholesterol: a meta-analysis of data from 170,000 participants in 26 randomised trials. Lancet.

[4] Silverman MG, Ference BA, Im K, Wiviott SD, Giugliano RP, Grundy SM (2016). Association between lowering LDL-C and cardiovascular risk reduction among different therapeutic interventions: a systematic review and meta-analysis. JAMA..

[5] Catapano AL, Graham I, De Backer G, Wiklund O, Chapman MJ, Drexel H (2016). 2016 ESC/EAS guidelines for the management of dyslipidaemias. Eur Heart J.

[6] Ference BA, Kastelein JJP, Ray KK, Ginsberg HN, Chapman MJ, Packard CJ (2019). Association of triglyceride-lowering LPL variants and LDL-C-lowering LDLR variants with risk of coronary heart disease. JAMA..

[7] Ohm J, Hjemdahl P, Skoglund PH, Discacciati A, Sundström J, Hambraeus K (2019). Lipid levels achieved after a first myocardial infarction and the prediction of recurrent atherosclerotic cardiovascular disease. Int J Cardiol.

[8] Calabrò P, Gragnano F (2019). Event recurrence after myocardial infarction: prediction is very difficult, especially about the future. Int J Cardiol.

[9] Schwartz GG, Abt M, Bao W, DeMicco D, Kallend D, Miller M (2015). Fasting triglycerides predict recurrent ischemic events in patients with acute coronary syndrome treated with statins. J Am Coll Cardiol.

[10] Miller M, Cannon CP, Murphy SA, Qin J, Ray KK, Braunwald E (2008). Impact of triglyceride levels beyond low-density lipoprotein cholesterol after acute coronary syndrome in the PROVE IT-TIMI 22 trial. J Am Coll Cardiol.

[11] Ishibashi S, Yamashita S, Arai H, Araki E, Yokote K, Suganami H (2016). Effects of K-877, a novel selective PPARα modulator (SPPARMα), in dyslipidaemic patients: a randomized, double blind, active- and placebo-controlled, phase 2 trial. Atherosclerosis..

[12] Ishibashi S, Arai H, Yokote K, Araki E, Suganami H, Yamashita S (2018). Efficacy and safety of pemafibrate (K-877), a selective peroxisome proliferator-activated receptor α modulator, in patients with dyslipidemia; results from a 24-week, randomized, double blind, active-controlled, phase 3 trial. J Clin Lipidol.

[13] Pradhan AD, Paynter NP, Everett BM, Glynn RJ, Amarenco P, Elam M (2018). Rationale and design of the pemafibrate to reduce cardiovascular outcomes by reducing triglycerides in patients with diabetes (PROMINENT) study. Am H J.

[14] Fruchart JC. Pemafibrate (K-877), a novel selective peroxisome proliferator-activated receptor alpha modulator for management of atherogenic dyslipidaemia. Cardiovasc Diabetol. 2017. 10.1186/s12933-017-0602-y. 10.1186/s12933-017-0602-y PMC5628452 28978316

[15] Yamamoto Y, Takei K, Arulmozhiraja A, Arulmozhiraja S, Sladek V, Matsuo N (2018). Molecular association model of PPARα and its new specific and efficient ligand, pemafibrate: structural basis for SPPARMα. Biochem Biophys Res Commun.

[16] Birjmohun RS, Hutten BA, Kastelein JJP, Stroes ESG (2005). Efficacy and safety of high-density lipoprotein cholesterol-increasing compounds: a meta-analysis of randomized controlled trials. J Am Coll Cardiol.

[17] Kraja AT, Province MA, Straka RJ, Ordovas JM, Borecki IB, Arnett DK (2010). Fenofibrate and metabolic syndrome. Endocr Metab Immune Disord Drug Targets.

[18] Vaziri ND (2006). Dyslipidemia of chronic renal failure: the nature, mechanisms, and potential consequences. Am J Physiol Ren Physiol.

[19] Mishima Y, Ando M, Kuyama A, Hisayama F, Ishioka T, Kibata M (1997). A simple method for identifying particle size of low-density lipoprotein using PAG electrophoresis: comparison between LipoPhor and LipoPrint LDL systems. J Jpn Atheroscler Soc.

[20] Imajo K, Hyogo H, Yoneda M, Honda Y, Kessoku T, Tomeno W (2014). LDL-migration index (LDL-MI), an indicator of small dense low-density lipoprotein (sdLDL), is higher in non-alcoholic steatohepatitis than in non-alcoholic fatty liver: a multicenter cross-sectional study. PLoS One.

[21] Miida T, Nishimura K, Hirayama S, Miyamoto Y, Nakamura M, Masuda D (2017). Homogeneous assays for LDL-C and HDL-C are reliable in both the postprandial and fasting state. J Atheroscler Thromb.

[22] Miwa K, Nakagawa K, Suzuki K, Inoue H (2001). Detection of the “midband” lipoprotein in patients with coronary artery spasm. Clin Cardiol.

[23] Wang H, Li H, Zhou Y, Liu J, Wang F, Zhao Q (2019). Pemafibrate tends to have better efficacy in treating dyslipidemia than fenofibrate. Curr Pharm Des.

[24] Araki E, Yamashita S, Arai H, Yokote K, Satoh J, Inoguchi T (2018). Effects of pemafibrate, a novel selective PPARα modulator, on lipid and glucose metabolism in patients with type 2 diabetes and hypertriglyceridemia: a randomized, double-blind, placebo-controlled, phase 3 trial. Diabetes Care.

[25] Sairyo M, Kobayashi T, Masuda D, Kanno K, Zhu Y, Okada T (2018). A novel selective PPARα modulator (SPPARMα), K-877 (pemafibrate), attenuates postprandial hypertriglyceridemia in mice. J Atheroscler Thromb.

[26] Hirose T, Teramoto T, Abe K, Taneyama T (2015). J-BENEFIT study group. Determinants of bezafibrate-induced improvements in LDL cholesterol in dyslipidemic patients with diabetes. J Atheroscler Thromb.

[27] Ginsberg HN, Le NA, Gibson JC (1985). Regulation of the production and catabolism of plasma low density lipoproteins in hypertriglyceridemic subjects; effect of weight loss. J Clin Invest.

[28] Sigurdsson G, Nicoll A, Lewis B (1975). Conversion of very low density lipoprotein to low density lipoprotein: a metabolic study of apolipoprotein B kinetics in human subjects. J Clin Invest.

[29] Schoonjans K, Staels B, Auwerx J (1996). The peroxisome proliferator activated receptors (PPARS) and their effects on lipid metabolism and adipocyte differentiation. Biochim Biophys Acta.

[30] Ai M, Otokozawa S, Asztalos BF, Ito Y, Nakajima K, White CC (2010). Small dense low density lipoprotein cholesterol and coronary heart disease: results from the Framingham offspring study. Clin Chem.

[31] Hoogeveen RC, Gaubatz JW, Sun W, Dodge RC, Crosby JR, Jiang J (2014). Small dense low-density lipoprotein-cholesterol concentrations predict risk for coronary heart disease: the atherosclerosis risk in communities (ARIC) study. Arterioscler Thromb Vasc Biol.

[32] Sakai K, Koba S, Nakamura Y, Yokota Y, Tsunoda F, Shoji M (2018). Small dense low-density lipoprotein cholesterol is a promising biomarker for secondary prevention in older men with stable coronary artery disease. Geriatr Gerontol Int.

[33] Hayashi T, Koba S, Ito Y, Hirano T (2017). Method for estimating high sdLDL-C by measuring triglyceride and apolipoprotein B levels. Lipids Health Dis.

[34] Cesaro A, Gragnano F, Fimiani F, Moscarella E, Diana V, Pariggiano I (2020). Impact of PCSK9 inhibitors on the quality of life of patients at high cardiovascular risk. Eur J Prev Cardiol.

[35] Farmaki P, Damaskos C, Garmpis N, Garmpi A, Savvanis S, Diamantis E (2020). PCSK9 inhibitors and cardiovascular disease: impact on cardiovascular outcomes. Curr Drug Discov Technol.

[36] Kjellmo CA, Hovland A, Lappegård KT (2018). CVD risk stratification in the PCSK9 era: is there a role for LDL subfractions?. Diseases..

[37] Ai M, Tanaka A, Tomie N, Ogita K, Sekine M, Numano F (2001). Triglyceride-rich lipoprotein cholesterol exceeds low-density lipoprotein cholesterol in hypertriglyceridemia patients. Horm Metab Res.

[38] Bezafibrate Infarction Prevention (BIP) Study (2000). Secondary prevention by raising HDL cholesterol and reducing triglycerides in patients with coronary artery disease. Circulation..

[39] Chapman MJ, Ginsberg HN, Amarenco P, Andreotti F, Borén J, Catapano AL (2011). Triglyceride-rich lipoproteins and high-density lipoprotein cholesterol in patients at high risk of cardiovascular disease: evidence and guidance for management. Eur Heart J.

[40] Sørensen LP, Andersen IR, Søndergaard E, Gormsen LC, Schmitz O, Christiansen JS (2011). Basal and insulin mediated VLDL-triglyceride kinetics in type 2 diabetic men. Diabetes..

[41] Joshi PH, Khokhar AA, Massaro JM, Lirette ST, Griswold ME, Martin SS, et al. Remnant lipoprotein cholesterol and incident coronary heart disease: the Jackson heart and Framingham offspring cohort studies. J Am Heart Assoc. 2016. 10.1161/JAHA.115.002765. 10.1161/JAHA.115.002765 PMC4889167 27130348

[42] Johansen RF, Søndergaard E, Sørensen LP, Jurik AG, Christiansen JS, Nielsen S (2016). Basal and insulin-regulated VLDL1 and VLDL2 kinetics in men with type 2 diabetes. Diabetologia..

[43] Noda H, Iso H, Irie F, Sairenchi T, Ohtaka E, Ohta H (2010). Association between non-high-density lipoprotein cholesterol concentrations and mortality from coronary heart disease among Japanese men and women: the Ibaraki prefectural health study. J Atheroscler Thromb.

[44] Robinson JG, Wang S, Smith BJ, Jacobson TA (2009). Meta-analysis of the relationship between non-high-density lipoprotein cholesterol reduction and coronary heart disease risk. J Am Coll Cardiol.

[45] Boot CS, Middling E, Allen J, Neely RD (2019). Evaluation of the non-HDL cholesterol to apolipoprotein B ratio as a screening test for dysbetalipoproteinemia. Clin Chem.

[46] Kameda K, Matsuzawa Y, Kubo M, Ishikawa K, Maejima I, Yamamura T (1984). Increased frequency of lipoprotein disorders similar to type III hyperlipoproteinemia in survivors of myocardial infarction. Atherosclerosis..

[47] Tsujita K, Sugiyama S, Sumida H, Shimomura H, Yamashita T, Yamanaga K (2015). Impact of dual lipid-lowering strategy with ezetimibe and atorvastatin on coronary plaque regression in patients with percutaneous coronary intervention: the multicenter randomized controlled PRECISE-IVUS trial. J Am Coll Cardiol.

